# True death toll of COVID-19 in Georgia: estimates of the number of deaths from COVID-19: Levan Kandelaki

**DOI:** 10.1093/eurpub/ckac131.062

**Published:** 2022-10-25

**Authors:** M Shakhnazarova

**Affiliations:** Medical Statistics, NCDC, Tbilisi, Georgia

## Abstract

**Problem:**

Georgia, in terms of Covid-19 attributed mortality, ranks 8th in the World.

**Description:**

In Georgia, there are no routinely conducted autopsies of the deceased. The identification of causes of death is based on the physician’s clinical decision. Absence of autopsies, lack of experience with Covid-19, are key issues of Covid-19 death certification. Considering COVID-19 as a primary cause of death led to over-reporting of COVID mortality among patients with severe comorbidities. This affects the identification of underlying cause of death (UCOD) and leads to misclassifications.

**Results:**

2021 mortal cases with Covid-19 as the UCOD, were analyzed. Cases were grouped by the interval between testing and death: <1 day - 170 cases (1.5%), 2-44 days - 9468 cases (83.5%), 45+ days - 1694 cases (15%). The group of cases with time intervals of 2-44 days, was sub-grouped according to causes mentioned in death certificates: A - only U07 is indicated, B - U07&any chain-of-event condition (pneumonia, respiratory distress, respiratory failure, etc.), C - U07& 1 or more significant contributing conditions (diabetes, stroke, etc.). According to the analysis in the group, 2-44 days distribution was: A - 6%; B - 81%; C - 13%. U07 cases in groups A and C without any respiratory conditions mentioned were defined as ‘unlikely’ COVID-19 death (19% of total). From groups with time intervals of < 1 and 45+ days (1864) 72 cases were randomly selected for in-depth analysis by a panel of experts, using additional medical records. Out of these cases, the panel of experts considered 80%, as ‘non-COVID-19’ deaths.

**Lessons:**

If ‘unlikely’ and ‘non-COVID-19’ deaths aren't counted, the number of deaths is reduced by 29.5%. Lack of duration of a disease represents a limitation. A similar revision of all/2-44 day’s interval COVID-19-related deaths is required to get a real number. Conduction of a verbal autopsy is necessary. Training on coding causes of death and existing regulations is important.

**Key messages:**

• There is an over-registration of COVID-19 as a UCOD.

• The lack of knowledge of death certification of cases with severe comorbidities and accidents leads to over estimates.

